# Current perspectives on the early identification and management of delirium after acute stroke reperfusion therapy

**DOI:** 10.3389/fneur.2025.1664070

**Published:** 2025-11-26

**Authors:** Ya-Nan Hao, Xiao-Yang Feng, Fei Wu, Xiao-Dan Yang, Xiao-Ling Zhang

**Affiliations:** 1 Department of Neurology, The Second Affiliated Hospital of Jiaxing University, Jiaxing, China; 2 Department of Neurology, Zhejiang Province People’s Hospital Haining Hospital, Jiaxing, China; 3 Jiaxing University Master Degree Cultivation Base, Zhejiang Chinese Medical University, Hangzhou, China; 4 Department of Hepatobiliary Surgery, The Second Affiliated Hospital of Jiaxing University, Jiaxing, China

**Keywords:** delirium after acute stroke reperfusion, risk factors, pathogenesis, biomarkers, intervention

## Abstract

Delirium is a common and severe complication following acute cerebral ischemic stroke reperfusion, affecting cognitive and motor recovery and associated with poor long-term outcomes, prolonged hospitalization, increased readmission, and higher mortality. Identifying risk factors, understanding the pathogenesis (especially reperfusion injury), and implementing early intervention are crucial. However, the complex etiology and limited efficacy of drug treatments make early detection and management challenging. Recent studies have identified factors such as age, NIHSS score, pre-stroke cognition, and post-reperfusion inflammation, along with biomarkers, as predictors of delirium. Family involvement and environmental optimization may also reduce risk. This review summarizes current evidence on risk factors, pathogenesis, biomarkers, and interventions to improve early identification, reduce disability, and improve prognosis.

## Introduction

Delirium is a frequently encountered acute or subacute neuropsychiatric syndrome in clinical settings, presenting with symptoms such as confusion, inattention, disorganized or incoherent thinking, and perceptual abnormalities that can vary significantly within a short timeframe. The latest International Classification of Diseases, 11th Edition (ICD-11), published by the World Health Organization, defines delirium as a syndrome characterized by the acute or subacute onset of attention disturbance (e.g., a reduced ability to direct, focus, sustain, and shift attention) and consciousness disturbance (e.g., impaired orientation to the environment). These symptoms, which often fluctuate within 24 h, are frequently accompanied by other cognitive deficits, including impairments in memory, language, visuospatial abilities, or perception. Delirium is commonly observed in hospitalized elderly patients and ICU patients. A recent meta-analysis of 35 studies reported a prevalence of 23.6% and incidence of 13.5% of delirium in hospitalized elderly individuals (1). In ICU patients aged 65 years or older, approximately 70% experience delirium (2). However, post-stroke delirium occurs at a rate of 18–30%, significantly impacting patient outcomes (3, 4).

With China’s aging population steadily increasing, stroke has emerged as a major public health concern, significantly affecting people’s health. Ischemic cerebral infarction, the most prevalent type of stroke, accounts for 69.6 to 70.8% of all strokes in China. Early reperfusion therapy (including intravenous thrombolysis and endovascular intervention) has proven to be the most effective treatment for ischemic stroke, but approximately 50% of patients still have a poor prognosis. Studies have shown that approximately 49.7% of patients with acute ischemic stroke who received reperfusion therapy develop delirium within the first 8 days of hospitalization (5). Recent studies on the relationship between reperfusion therapy and delirium have increased, primarily focusing on whether reperfusion therapy reduces delirium risk by improving brain ischemia, or potentially increases delirium incidence due to treatment itself (e.g., surgical stress). Research by Czyzycki et al. suggests that reperfusion therapy (including thrombolysis and mechanical thrombectomy) may have potential in preventing delirium by improving brain injury. However, the effect is influenced by pre-stroke cognitive status, with lower delirium rates observed in those without cognitive decline prior to the stroke (6). On the other hand, some studies have found a significant association between mechanical thrombectomy and postoperative delirium, particularly in elderly patients, which may be related to surgical trauma and perioperative management factors (7, 8).

The relationship between reperfusion and delirium is still unclear, and current interventions are limited. There is a lack of comprehensive research on delirium after acute cerebral ischemic stroke reperfusion in China, which is inconsistent with the large number of stroke patients in the country. This review integrates the latest domestic and international studies to summarize the potential pathogenesis, risk factors, biomarkers, and interventions for delirium following reperfusion in acute stroke, aiming to improve early identification, timely intervention, and overall prognosis.

## Methods

This narrative review was conducted based on a comprehensive literature search of PubMed, Web of Science, MEDLINE, EMBASE, and the Cochrane Library for relevant English-language articles published up to June 2025. The search used key words such as “acute stroke reperfusion therapy,” “delirium.” The Boolean combinations of search terms were (acute ischemic stroke OR acute cerebral infarction OR acute stroke) AND (reperfusion therapy OR intravenous thrombolysis OR mechanical thrombectomy) AND (delirium OR cognitive dysfunction OR altered mental status) NOT (pediatric stroke OR case reports). The research types included randomized controlled trials, observational studies and authoritative guidelines. The inclusion criteria were the studies include the occurrence of delirium during reperfusion therapy (intravenous thrombolysis, mechanical thrombectomy) for acute ischemic stroke, as well as the incidence, pathogenesis, assessment methods and treatment approaches of post-stroke reperfusion delirium. The exclusion criteria were pediatric stroke and case reports. Based on a clear research scope, studies focusing on acute ischemic stroke reperfusion therapy and exploring delirium risk factors, assessment tools, or management strategies were prioritized. The review emphasized high-quality, methodologically rigorous evidence, critically evaluating existing views and identifying gaps in the pathogenesis and management of delirium after reperfusion. It also suggested future research directions, including imaging and biological markers for delirium identification.

### Pathogenesis of delirium after acute stroke

Although delirium arises from diverse etiologies, its clinical manifestations are often similar, implying a shared underlying pathogenesis. However, the precise mechanisms of delirium remain incompletely understood. Most scholars believe that the occurrence of delirium after acute stroke reperfusion therapy may be related to multiple pathophysiological mechanisms. The primary hypotheses are as follows:

#### Neuroinflammatory cascade reaction hypothesis

Although reperfusion therapy can restore cerebral blood flow, the local inflammatory response caused by ischemic brain injury may lead to blood–brain barrier damage, triggering neuroinflammation. The inflammatory response occurs throughout the ischemic injury process and is one of the possible mechanisms leading to delirium after reperfusion therapy. Increased inflammatory response in circulation is positively correlated with the development of delirium. Some inflammatory biomarkers (such as inflammatory factors NFL, IL-6/8 /10/1β and TNF-*α*) have been identified as risk factors for delirium in elderly individuals (9, 10). Increasing evidence suggests that glial cell dysfunction is associated with delirium (11). Research by Witcher KG et al. found that microglia, as key mediators of chronic inflammation, not only respond to and mediate inflammatory signals, causing brain tissue damage and blood–brain barrier disruption, but also influence neuronal responses to traumatic brain injury (12). A study by Liu J et al. found that midbrain astrocyte-derived neurotrophic factor (MANF) may reduce the expression of IL-1β, IL-6, and TNF-*α* in the serum and cerebrospinal fluid of mice induced by surgery, thereby inhibiting surgery-induced inflammation, microglia activation, and M1 polarization, protecting mice from delirium-like behavioral disorders (13). However, a decrease in anti-inflammatory factors may exacerbate neuroinflammatory-related systemic damage.

To summarize, Ischemia–reperfusion injury activates microglia and astrocytes, triggering the release of pro-inflammatory factors like IL-1β and TNF-*α*, leading to neuroinflammation. This inflammation damages neurons, disrupts the blood–brain barrier, and enhances immune cell infiltration, further amplifying the inflammatory response (14). It also impairs cholinergic function and promotes glutamate excitotoxicity, disrupting neural circuits and contributing to delirium.

#### Oxidative stress and neurotoxicity hypothesis

Compared to other organs, the brain has weaker antioxidant defenses, making it more vulnerable to oxidative damage. During brain ischemia, reduced oxygen supply leads to mitochondrial dysfunction, metabolic disturbances (decreased ATP), and ion pump failure, causing elevated intracellular calcium levels and promoting free radical production. Reperfusion rapidly restores oxygen, converting it into reactive oxygen species (ROS) like superoxide, hydrogen peroxide, and hydroxyl radicals. These ROS react with intracellular lipids, proteins, and DNA, causing cell membrane rupture, protein denaturation, and DNA damage, leading to brain cell death and blood–brain barrier disruption, which in turn exacerbates brain injury. Increased barrier permeability allows plasma proteins like fibrinogen to leak into the brain, triggering microglial activation and a neurotoxic environment (15). Blood products from hemorrhagic transformation (e.g., iron ions) induce oxidative stress and neuronal apoptosis, especially in the cortex, raising delirium risk (16). Microcirculatory disorders after reperfusion cause local hypoxia, worsening mitochondrial dysfunction and energy failure. However, Research by Sun et al. found that remote ischemic preconditioning reduces oxidative stress and inflammation through the Nrf2/HO-1 pathway, improving neurological function (17).

#### Abnormal cerebral perfusion and neurotransmitter imbalance hypothesis

Reperfusion therapy restores blood flow but can cause brain perfusion imbalance, impairing vascular autoregulation and leading to blood flow fluctuations, disrupting neuronal metabolism and neurotransmitter balance. Reperfusion therapy may disrupt neurotransmitters in the brain, such as acetylcholine, dopamine, histamine, norepinephrine, Glutamate, *γ*-aminobutyric acid, aspartate and serotonin and this imbalance is considered a key mechanism for post-stroke delirium (18). Animal studies have shown that interference with multiple neurotransmitter systems can produce features of delirium (19). Acetylcholine (ACh) receptor antagonists cause widespread EEG slowing, most notably an increased *δ* wave frequency (1–3 Hz) and decreased *α* wave frequency (8–12 Hz), which is associated with delirium (20). Research by Massoudi N found that cholinesterase inhibitors are beneficial in improving post-stroke delirium symptoms (21). Acute cholinergic dysfunction can trigger delirium and may largely contribute to delirium symptoms, although this effect is most likely to occur in patients (or animals) with pre-existing cholinergic vulnerability (22). Antipsychotic medications, primarily acting through the blockade of D2 dopamine receptors, have long been used in the treatment of delirium. However, numerous prevention and treatment trials have failed to demonstrate the benefits of various antipsychotics (including haloperidol, olanzapine, or quetiapine) in preventing or treating delirium in multiple settings (23, 24), the hyperdopaminergic theory of delirium has been significantly weakened. Therefore, while dopaminergic status may influence the psychomotor state during delirium, it is unlikely to play a mechanistic role at the syndrome level. Histamine affects arousal by activating projections from the hypothalamus to the prefrontal cortex (PFC), limbic system, and basal ganglia. Several studies have highlighted the importance of histamine in wakefulness and alertness (25). Although clinical studies have not specifically focused on delirium, first-generation antihistamines (H1 receptor antagonists) have been widely described as having sedative effects, reducing the brain’s arousal state, while delirium is a known adverse effect of both H1 and H2 receptor antagonists (26). Norepinephrine has a profound impact on prefrontal cortex (PFC) activity. Norepinephrine neurons in the locus coeruleus are silent during rapid eye movement (REM) sleep but exhibit significant phasic firing during wakeful alertness. In contrast, under stress, activation of the amygdala stimulates the locus coeruleus, triggering high-toned norepinephrine activity, which leads to poor attention performance (27). In this scenario, cognitive and behavioral functions shift from the prefrontal cortex’s thoughtful “top-down” regulation to more reflexive, emotion-driven responses driven by the amygdala (e.g., fear and threat). Therefore, both excessive and insufficient norepinephrine activity can impair prefrontal cortex function (28), and it can be speculated that these different states of arousal may contribute to the high and low activity states seen in delirium.

#### Neural network disconnection hypothesis

The effect of neurotransmitters on brain states depends on the neural anatomical networks they act upon. Research by Morandi A et al. showed that loss of integrity in the corpus callosum between the hemispheres is associated with prolonged delirium duration (29). Diffusion tensor imaging revealed abnormalities in regions such as the hippocampus, thalamus, basal ganglia, and cerebellum (as well as related white matter tracts: fiber bundles, fornix, internal capsule, and corpus callosum), with these abnormalities linked to the incidence and severity of delirium (30). Although most research on cerebral ischemia–reperfusion injury focuses on cortical tissue, studies have shown that in the Middle cerebral artery occlusion (MCAO) model, hippocampal neurons are also damaged as neurological function worsens. Gao et al. reported that synaptic connection strength and protein expression are inhibited, with impaired synaptic plasticity being a key mechanism of reperfusion injury (31).

#### Reperfusion-specific injury mechanism hypothesis

Mechanical thrombectomy may cause endothelial damage and plaque detachment, leading to embolism or reocclusion. Repeated ischemia–reperfusion worsens oxidative stress and mitochondrial damage, triggering neuronal necrosis (32). Anesthetics like propofol may impair cortical function, worsening postoperative delirium (33).

**Figure 1:**
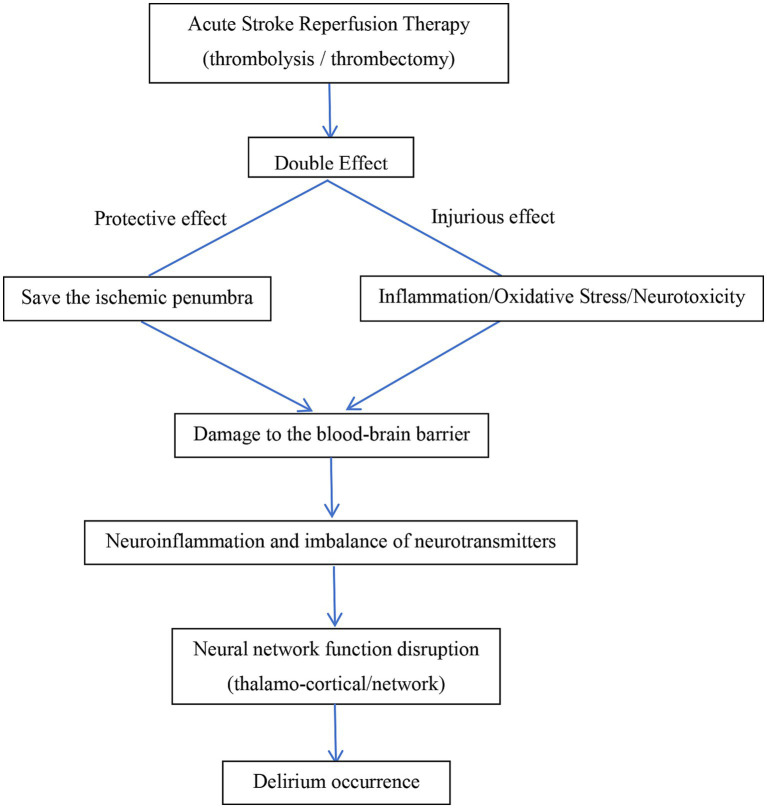
Mechanisms of delirium occurrence after acute stroke reperfusion therapy.

In summary, the occurrence of delirium after reperfusion is the result of multiple mechanisms working together. Key factors include perfusion imbalance and reperfusion-specific damage leading to neuroinflammation, oxidative stress, neurotoxicity, and blood–brain barrier disruption. These ultimately cause neurotransmitter regulation and neural network connectivity impairments, resulting in system integration failure and delirium symptoms. Future research should focus on precise therapeutic strategies targeting inflammatory pathways and blood–brain barrier protection. The possible pathological mechanisms of delirium occurrence after acute stroke reperfusion therapy are shown in Figure 1.

### Risk factors for delirium after acute stroke reperfusion therapy

Most scholars, both domestically and internationally, agree that the onset of delirium is caused by a combination of factors, including both predisposing and precipitating risk factors. Predisposing factors include advanced age, cognitive impairment, comorbidities such as cardiovascular or renal diseases, a history of mental disorders, alcohol abuse, malnutrition, and visual or hearing impairments (34, 35). Neuroimaging studies have also demonstrated that patients with brain atrophy or white matter lesions are at a higher risk of developing delirium (36). Precipitating factors encompass traumatic brain injury, stroke, hypoxia, metabolic disorders, infections, trauma, surgery, anesthesia, pain, physical restraints, and emotional distress. Additionally, some studies have shown that certain medications, such as opioids and sedative-hypnotics, may increase the incidence of delirium (37). However, family involvement can significantly reduce the occurrence of delirium in critically ill patients (38).

In addition to the factors mentioned above, the location, size, number, and severity of strokes may also influence the occurrence of acute post-stroke delirium. Infarction in key areas (such as the left middle frontal gyrus, left angular gyrus, left basal ganglia, and left anterior thalamus) and certain conduction tracts (such as the right corticospinal tract, left posterior inferior cerebellar tract, left arcuate fasciculus, and left basal ganglia peripheral white matter), as well as asymptomatic cerebrovascular diseases (such as white matter lesions and microbleeds), are risk factors for delirium after stroke. Right hemisphere infarction is a risk factor for spatial neglect, which in turn is associated with delirium after stroke (39, 40). Patients with supratentorial, anterior circulation, or cortical lesions, higher NIHSS scores, cardioembolic or large artery atherosclerotic etiologies, intracranial hemorrhage, or larger/more extensive infarcts are more likely to develop delirium (41–43).

Reperfusion therapy (e.g., thrombolysis) is linked to a reduced risk of delirium, while the effect of mechanical thrombectomy is inconsistent and depends on patient characteristics. Delirium is associated with worse outcomes (e.g., mortality and disability) in reperfusion patients, though therapy may reduce delirium by improving brain perfusion (44). Successful reperfusion may also help reduce delirium-related complications (45). The impact of reperfusion therapy varies based on factors like timing, age, and comorbidities, requiring individualized evaluation of its benefits for delirium.

The possible risk factors for the occurrence of delirium after reperfusion therapy for acute stroke, including susceptibility factors, inducing factors and stroke-related factors were showed in Table 1.

**Table 1:** Risk factors for delirium after stroke reperfusion.

Susceptibility factors	Precipitating factors	Stroke-related factors
High age, Pre-stroke cognitive impairment or psychiatric history, Malnutrition, Alcohol use, Visual or hearing impairment, Comorbid severe cardiovascular, hepatic, renal, or pulmonary diseases, Cerebral atrophy and widespread white matter lesions.	Hypoxia, Metabolic disturbances, Infection, Trauma, Surgery, Anesthetic or sedative Drugs, Pain, Physical restraints, Indwelling catheters (IV, NG, urinary), Sleep deprivation, Frequent nighttime care, Emotional stress.	Location of the infarct (supratentorial, anterior circulation, cortical lesions), Multiple asymptomatic white matter lesions or microbleeds within the brain, Reperfusion injury (e.g., hemorrhagic transformation), Mechanical thrombectomy (surgical trauma, anesthetic drugs, intraoperative embolus dislodgement, hemodynamic fluctuations, etc.), Stroke etiology (e.g., cardioembolic, large artery atherosclerotic), The number of strokes, NIHSS scores, The presence of hemorrhagic conversion

Susceptibility factors: the fundamental factors that make an individual more prone to delirium, usually related to the individual’s health condition, age, psychological state, or past medical history, etc. Triggering factors: the specific external or environmental factors that may trigger the occurrence of delirium during acute events or treatment processes. IV, Intravenous; NG, Nasogastric; NIHSS, National Institutes of Health Stroke Scale.

In conclusion, reperfusion therapy has a complex bidirectional effect on delirium. On one hand, timely and effective reperfusion can restore blood flow, limit ischemic penumbra expansion, improve neurological outcomes, and reduce delirium incidence by enhancing brain plasticity. On the other hand, reperfusion can trigger inflammatory responses and oxidative stress, leading to reperfusion injury and worsening brain dysfunction. Invasive procedures like thrombectomy may also increase delirium risk through anesthesia, hemodynamic fluctuations, and microemboli. Therefore, clinical practice must strike a dynamic balance between maximizing the benefits of reperfusion and minimizing the risk of delirium. This requires optimizing the timing and strategy of reperfusion, enhancing perioperative monitoring, precisely controlling blood flow parameters, and identifying high-risk individuals, thereby improving neurological recovery while reducing the occurrence of delirium.

### Assessment tools for delirium after acute stroke

Symptoms such as speech disturbances and apathy in patients with acute stroke reperfusion therapy can be easily mistaken for delirium symptoms, complicating the screening and diagnosis process. Consequently, utilizing appropriate assessment tools is crucial for detecting delirium after acute stroke reperfusion therapy. Among the commonly used delirium screening and assessment tools is the Confusion Assessment Method (CAM), which is widely regarded as the most effective screening tool both domestically and internationally (46). CAM has been translated into over 20 languages. It assesses four features of delirium: (1) Acute onset and fluctuating changes in mental state; (2) Difficulty concentrating; (3) Disorganized thinking; (4) Altered level of consciousness. To meet the diagnostic criteria, the patient must exhibit both (1) and (2), along with at least one of (3) or (4) CAM has been adapted into other scales through extensive clinical application. For example, the Chinese version of the 3-Minute Diagnostic Interview for CAM-defined Delirium (3D-CAM), translated and revised by Gao et al. in 2018, simplifies the assessment of the four CAM features, significantly reducing the time required for evaluation. The study also assessed the sensitivity and specificity of the 3D-CAM, which were found to be 94.73 and 97.92%, respectively (47). However, this scale has a limitation, which is that it cannot be used for patients in deep sedation or coma. The Confusion Assessment Method for the Intensive Care Unit (CAM-ICU), modified by Ely et al., is primarily used to assess delirium in patients admitted in ICU and is appropriate for situations where patients cannot cooperate verbally (48). It is convenient, quick, and accurate, and is frequently regarded as the diagnostic “gold standard” by ICU healthcare professionals.

In 2014, Bellelli et al. introduced a new delirium assessment tool, the 4A Test (4 “A”s Test, 4AT) (49). This scale assesses four key aspects: alertness, orientation, attention, and acute changes or fluctuations in mental status. It is simple and easy to use, enabling clinical staff to administer it without the need for specialized training. A score of ≥ 4 suggests the potential presence of delirium, with or without cognitive impairment; a score of 1 to 3 indicates possible cognitive impairment; and a score of 0 suggests no delirium or severe cognitive impairment. The scale’s sensitivity is 90%, and its specificity is 94%, as validated by Lai YH et al. (50). Huang et al. were the first to translate the Delirium Rating Scale, revised in 1998 (DRS-R-98), into Chinese (51). This scale consists of 16 items and typically takes around 30 min to complete. It covers multiple areas, including sleep, hallucinations, emotions, attention, and visuospatial abilities. The scale has been validated with a sensitivity of 89.3% and a specificity of 96.8%, and it is now widely used in clinical practice.

The Nursing Delirium Screening Scale (Nu-DESC) is a nurse-administered delirium screening tool suitable for hospitalized patients, with a sensitivity of 29–95% and a specificity of 69–90%. However, it is not applicable to patients with endotracheal intubation (52). The Intensive Care Delirium Screening Checklist (ICDSC) is a delirium screening tool specifically designed for ICU patients, applicable to both hospitalized and ICU settings, with a sensitivity of 74–99% and a specificity of 64–82%. However, it requires longer scoring time and higher training for non-specialists (53). The diagnostic criteria, user groups and limitations of common assessment scales for delirium after reperfusion in acute stroke were presented in Table 2.

**Table 2:** Delirium assessment scales after acute stroke reperfusion.

Tool	Diagnostic criteria	Users	Limitations
CAM	Acute onset and fluctuating course; inattention; Disorganized thinking; Altered level of consciousness. Positive scores for criteria 1 AND 2, as well as either 3 OR 4	Inpatients, especially elderly patients, postoperative patients, and general medical wards.	Inter-rater reliability is variable; limited applicability in children and patients with severe language barriers.
CAM- ICU	Sudden change in mental status; Inattention; Disorganized thinking; Change in consciousness level. Positive scores for criteria 1 AND 2, as well as either 3 OR 4.	Patients in the ICU, particularly those on mechanical ventilation or unable to communicate verbally.	Lower sensitivity for mild delirium; requires combination with other tools for accurate diagnosis.
DRS-R-98	13-item severity scale (each scored 0–3) plus a 3-item diagnostic scale (each scored 0–2); domains address orientation, psychomotor status, sleep, perceptual disorders, delusions, mood, thought rocess, and cognition (attention, memory, visuospatial) Total score ranges from 0 to 46, Positive scores for ≥15.	Inpatients, ICU patients, and postoperative patients.	Time-consuming to administer; requires trained personnel for accurate interpretation.
4AT	item test including alertness (4 points), orientation (1–2 points), attention (1–2 points), acute change and fluctuations (4 points) Positive scores for ≥4.	Inpatients, postoperative patients.	Simplified diagnostic criteria may miss some key features of delirium.
Nu-DESC	8 items assessing nursing staff’s ability to detect delirium. Total score ranges from 1 to 8, with ≥3 indicating a positive result.	Nurses assessing delirium in hospitalized patients.	Ineffective for patients on mechanical ventilation or under sedation.
ICDSC	8 items assessing changes in consciousness, attention, orientation, hallucinations, agitation, inappropriate speech, sleep–wake cycle disturbances, and symptom fluctuations. Total score ranges from 1 to 8, with ≥4 indicating a positive result.	ICU patients, hospitalized patients.	Administer; requires trained personnel for accurate interpretation.

CAM, Confusion Assess ment Method; CAM-ICU, Confusion Assess ment Method for the Intensive Care Unit; ICU, Intensive Care Unit; DRS-R-98, Delirium Rating Scale Revised-98; 4AT, Assessment Test for Delirium and Cognitive Impairment; Nu-DESC, Nursing Delirium Screening Scale; ICDSC, Intensive Care Delirium Screening Checklist. This table showed the diagnostic criteria, user groups and limitations of common assessment scales for delirium after reperfusion in acute stroke.

Due to limitations in scale usage, such as patients’ consciousness and assessors’ subjective judgment, recent studies suggest using CT or MRI neuroimaging to assess post-stroke reperfusion delirium. The following markers have demonstrated certain value in prospective studies and have shown good reproducibility and feasibility in the stroke unit.

#### MTA score

The Pathological hippocampal Medial Temporal Lobe Atrophy (MTA) score on CT, by quantifying the pathological changes (atrophy, low-density lesions, etc.) in the hippocampal region, assists in predicting the risk of delirium (54). This score demonstrated good reproducibility in a multicenter study, was simple to operate, and was suitable for routine application in stroke units.

#### Fazekas score

The Fazekas score for pathological leukoaraiosis on cranial MRI is used to assess the severity of brain white matter lesions. Studies have shown that a higher Fazekas score is significantly associated with the occurrence of post-reperfusion delirium (55). This score demonstrates certain stability across different devices and scanning parameters, and is widely used in clinical practice. It can be regarded as a routine indicator for assessing the risk of post-stroke reperfusion delirium.

#### ASL

The Arterial Spin Labeling (ASL) can non-invasively assess cerebral blood flow perfusion and detect the areas of low perfusion in the brain after reperfusion. Multiple prospective studies have confirmed that the abnormal cerebral blood flow shown by ASL is closely related to the occurrence of delirium (56). Its advantages lie in the fact that no contrast agent is required, it is highly safe, and it can be easily implemented on the multimodal imaging platform of the stroke unit, with good repeatability.

#### DTI

The Diffusion Tensor Imaging (DTI) can reflect the integrity and direction of nerve fibers and is used to assess nerve network damage. The research has found that patients with post-reperfusion delirium exhibit abnormal diffusion of white matter fiber tracts (57). With the advancement of technology, the application of DTI in stroke units has gradually become widespread, and its results have high reproducibility.

#### SWI

The Susceptibility-Weighted Imaging (SWI) is sensitive to microbleeds, vascular malformations, and other minute lesions, and can detect potential microbleeds or vascular abnormalities after reperfusion. These lesions may be related to the mechanism of delirium (58). SWI can produce stable imaging on MRI devices of different magnetic field strengths, with high image resolution, which helps stroke units accurately identify high-risk individuals.

The above biomarkers suggest that neurofunctional imaging and other imaging techniques may be an important direction for the future development of delirium assessment.

### Biomarkers associated with delirium after acute stroke reperfusion

Some biomarkers can predict the risk of delirium after stroke, such as inflammatory factors: neutrophil-to-lymphocyte ratio (NLR), C-reactive protein (CRP), IL-6/8/10/1β, and TNF-*α* (59–61); neurofilament light chain (NFL) (62); and neuroglial cells, including astrocytes, microglia, and neuroglial activation factors (e.g., S100β) (63); and neuroprotective factors such as insulin-like growth factor 1 (IGF-1) (64, 65). Bai Y et al. found that the brain’s response to transcranial magnetic stimulation electroencephalography (TMS-EEG) can identify high-risk patients for delirium after stroke (66).

### Treatment of delirium after acute stroke reperfusion therapy

The pathogenesis of post-stroke reperfusion delirium involves multiple pathological mechanisms, with numerous triggering and risk factors. Currently, there are no unified guidelines for the treatment, both domestically and internationally.

Several systematic reviews have evaluated the treatment of delirium in hospitalized patients, concluding that antipsychotic medications do not significantly affect the severity of delirium, symptom relief, or mortality and should not be the primary treatment approach (67–69). Thus, the prevailing opinion among domestic experts is that post-stroke reperfusion delirium treatment should focus primarily on addressing precipitating factors, with non-pharmacological interventions being prioritized. Antipsychotic medications may be used to treat delirium with behavioral and emotional disturbances when it causes significant distress, poses a safety risk, or interferes with essential treatments, and non-drug therapies fail. Recommended drugs include haloperidol, quetiapine, olanzapine, and risperidone, starting at low doses and gradually increasing based on improvement and side effects, with treatment lasting 1–2 weeks. Medication can be tapered after delirium resolves for 2 days. During the medication period, it is necessary to monitor for extrapyramidal adverse reactions, electrocardiogram, changes in QT interval and changes in consciousness level. Table 3 showed the treatment strategies for delirium occurrence after reperfusion therapy for acute stroke, including non-pharmacological treatment and pharmacological treatment. Research on delirium treatment/prevention in acute stroke patients receiving reperfusion therapy is limited. This article provides recommendations for non-pharmacological prevention strategies based on existing literature (70). Non-pharmaceutical treatments and specific operations were presented in Table 4.

**Table 3:** Treatment of delirium after acute stroke reperfusion.

Non-pharmacological treatment	Pharmacological treatment
Repositioning (orientation through conversation, placement of accurate calendars and clocks); Cognitive stimulation (dialog, psychological exercises, therapeutic activities); Sleep hygiene (avoiding excessive daytime sleep, promoting nighttime sleep, relaxation activities before bedtime, minimizing external noise, appropriate nighttime lighting, and avoiding nighttime medication administration); Early mobilization (providing safe transfers, opportunities for movement and exercise); Reducing catheter use (intravenous catheters, nasogastric tubes, urinary catheters); Reducing Restraints and Bed Alarms; Enhancing sensory function (use of glasses, hearing aids, and other assistive devices); Adequate hydration and nutrition (timely identification of dehydration and dysphagia risks, encouragement of oral intake); Intestinal and bladder hygiene (early ambulation to the bathroom, early identification and treatment of urinary retention and constipation); Family and caregiver involvement (placing familiar objects and photographs at the bedside);	Antipsychotics (haloperidol, quetiapine, risperidone, olanzapine, etc.); Benzodiazepines (diazepam, lorazepam, etc. only used for delirium caused by by benzodiazepine withdrawal or alcohol withdrawal); Sedatives (propofol, dexmedetomidine, etc.only for patients with delirium who require mechanical ventilation in a critical care setting)

The treatment strategies for delirium after acute stroke reperfusion draw on those for delirium, including non-pharmacological treatment and pharmacological treatment.

**Table 4:** Non-pharmacological treatment strategies for delirium after acute stroke reperfusion.

Non-pharmacological treatment	Examples of methods
Environment	The environment is bright, the signs are clear, and the calendars and clocks are placed correctly.
Cognitive Stimulation	Directed conversation, introduction of the environment and staff members, placing family members’ or memorial photos beside the bed, encouraging patients to play brain-training games.
The involvement of family members or close relatives	Encourage family members to provide care and have relatives or friends visit.
Communication among medical staff	For patients with aphasia or those on mechanical ventilation, it is recommended that medical staff use the shift report book for communication, promptly identify and record any changes in the patient’s consciousness/thinking, and conduct a detailed shift handover.
Sleep Hygiene	Avoid excessive daytime sleep and cultivate good nighttime sleep habits, such as relaxation activities before sleep, avoiding external noises, appropriate nighttime lighting, and avoiding nighttime medication administration, etc.
Early Mobilization	At least 2 activities per day are recommended, and the intensity can be adjusted according to the specific condition; Patients who can walk are encouraged to get out of bed and move as soon as possible; Patients who cannot walk are encouraged to perform passive exercises and start physical and occupational rehabilitation exercises as soon as possible.
Catheter / Constraint	When the condition permits, remove the intravenous catheter, urinary catheter, body restraints and other fixation devices as soon as possible.
Pain Management	Repeated pain screening; Provide multimodal treatment, including postural adjustment, physical therapy/occupational therapy, and neurological/psychological therapy, if necessary.
Drug Review	Evaluate all therapeutic drugs, reduce the number of medications, and avoid those that may exacerbate delirium.
Enhancing Sensory Capabilities	Provide glasses, hearing aids, etc. if necessary.
Abundant Water and Nutrients	Timely identify the risks of dehydration and dysphagia, encourage patients to drink plenty of water, maintain a balanced diet, and avoid aspiration.

Reperfusion therapy for acute stroke is prone to induce delirium. The non-pharmaceutical treatment methods listed in this table may reduce the occurrence of delirium after reperfusion to a certain extent.

In summary, the difficulty in identifying delirium after acute stroke reperfusion can be attributed to the following factors:

1. Insufficient understanding of post-stroke delirium, leading to the perception that elderly patients’ drowsiness or confusion after illness is a normal phenomenon;
2. Delirium is often mistaken for a symptom of stroke, rather than recognized as a distinct clinical entity;
3. Inadequate familiarity with delirium screening tools, resulting in low diagnostic rates;
4. Delirium is frequently overlooked during discharge, transfer, or follow-up;
5. Poor communication among nursing staff when dealing with patients experiencing mental confusion, delaying the recognition of changes in consciousness or cognition.

## Conclusion

Delirium is a common and serious complication after acute stroke reperfusion therapy. Its symptoms overlap with stroke symptoms, making diagnosis difficult. Traditional scales have limitations, such as the patient’s consciousness state and the subjectivity of the assessor. Some neuroimaging and serum biomarkers now serve as effective tools for early detection of delirium after stroke reperfusion. While the exact mechanism remains unclear, evidence suggests links to neuroinflammation, oxidative stress, and neurotransmitter imbalance. Despite poor prognosis, no effective treatment exists, and behavioral interventions are critical for prevention. Future research is needed to develop better strategies for this condition. Standardized treatment in stroke units is key to prevention. Future research is needed to clarify its pathogenesis and improve prevention and treatment.
